# The effects of heat and freeze-thaw cycling on naloxone stability

**DOI:** 10.1186/s12954-019-0288-4

**Published:** 2019-02-27

**Authors:** Dulcie Lai, Amy Trinh Pham, Praveen P. Nekkar Rao, Michael A. Beazely

**Affiliations:** School of Pharmacy, Faculty of Science, University of Waterloo, 10 Victoria Street South, Kitchener, Ontario N2G 1C5 Canada

**Keywords:** Naloxone, Naloxone stability, Opioid overdose, Harm reduction

## Abstract

**Purpose:**

The availability of take home naloxone (THN) was increased for Canadians in 2016, including access to kits via pharmacies. Unlike typical over-the-counter (OTC) and prescription drugs, THN kits may be stored in non-standard conditions, including in vehicles, backpacks, and out of doors. To evaluate whether these non-standard storage conditions affect stability, we investigated the impact of heat and freeze-thaw cycling on naloxone hydrochloride stability.

**Methods:**

To assess the effect of heat, naloxone hydrochloride ampoules were exposed to 80 °C in a temperature-controlled oven for 8 h followed by 16 h at room temperature. To assess the effect of freeze-thaw cycles, naloxone hydrochloride ampoules were exposed to − 20 °C for 16 h followed by 8 h at 4 °C. The impact of these conditions on naloxone hydrochloride stability was evaluated each day for 1 week and after 2 and 4 weeks. The concentration of remaining naloxone hydrochloride was quantified using high-performance liquid chromatography (HPLC). Naloxone hydrochloride ampoules stored at room temperature served as the experimental control.

**Results:**

Naloxone hydrochloride ampoules exhibit no changes in drug concentration following exposure to heat or freeze-thaw cycles for up to 28 days compared to ampoules maintained at room temperature (as indicated in the product monograph).

**Conclusions:**

Naloxone hydrochloride remains chemically stable following exposure to heat or freeze-thaw cycles after 28 days. If THN kits are stored in non-standard conditions (for up to 28 days) the active naloxone is likely to remain stable. Despite this, pharmacists should continue to emphasize the importance of appropriate storage of THN kits to ensure optimal efficacy should naloxone administration be required in an emergency situation.

**Electronic supplementary material:**

The online version of this article (10.1186/s12954-019-0288-4) contains supplementary material, which is available to authorized users.

## Introduction

In North America, opioid-related overdose is the leading cause of death in individuals between the ages of 18 and 35 [1–3]. In 2016, there were 2816 opioid-related deaths in Canada with approximately one third occurring in Ontario [4, 5]. Since 2003, the rate of opioid-related deaths has more than doubled [5]. In response to this crisis, prescription requirements for naloxone, an opioid antagonist used to reverse opioid-overdoses, were modified in 2016 by Health Canada and the National Association of Pharmacy Regulatory Authorities (NAPRA) [6–8]. These changes permitted Canadians to access to THN without a prescription and also expanded the description of who is eligible to receive a naloxone kit [6–8]. Currently, several jurisdictions in Canada, including Alberta, Ontario, Northwest Territories, and Yukon, offer unrestricted access to THN to anyone at risk of an opioid overdose or those in a position to assist in an opioid-related overdose [9].

The availability of THN outside of healthcare settings raises concerns regarding naloxone stability when exposed to conditions outside of the 15–30 °C range as indicated in the product monograph [10]. For example, individuals often inquire about the possibility of THN kit storage within their vehicles for accessibility (personal communication). Storage of THN kits outside or in vehicles may be problematic due to extreme and fluctuating temperatures among the seasons within Canada, which may impact naloxone stability and therefore efficacy. Expiration date and storage conditions are determined by satisfying the International Conference on Harmonization of Technical Requirements for Registration of Pharmaceuticals for Human Use (ICH) Guidelines for Stability Testing of New Drug Substances and Products [11]. These guidelines, however, do not address unconventional conditions (e.g., temperature extremes or freeze-thaw cycles). The findings of this study intend to provide healthcare providers, specifically pharmacists, with practical information to relay to individuals with THN kits who may have deviated from the recommended storage conditions.

Previous studies have investigated the consequence of naloxone storage under unconventional conditions. However, they are limited by the fact that they do not reflect conditions that may be encountered if THN kits were left, either intentionally or unintentionally, within a vehicle. For example, although temperatures as high as 70 °C have been evaluated, internal vehicle temperatures can reach up to 80 °C [12, 13]. Several studies also assessed the effect of temperature fluctuations from extreme cold to heat (− 20 °C to 70 °C; − 6 °C to 54 °C; − 20 °C to 45 °C); however, it is unlikely that these conditions would be experienced within the span of a single day, should a THN kit be left within a vehicle [12, 14, 15]. These studies also did not investigate the association of stability with the duration of exposure. Therefore, the objective of this study was to identify how daily exposure to heat (mimicking vehicle storage in the summer months) or freeze-thaw cycles (mimicking vehicle/outdoor storage in the winter months) may impact the stability of naloxone hydrochloride for up to 4 weeks. Findings of this study intend to provide pharmacists with additional counseling points should THN kits be stored outside of the recommended conditions.

## Methods

### Materials

Naloxone hydrochloride ampoules (1 mL) were obtained from Sandoz (0.4 mg/mL; Lot # GH3080; Exp: 2019-03). Naloxone hydrochloride reference standard was purchased from Cayman Chemical (Ann Arbor, MI; CAS: 357-08-4; Item No. 15594). HPLC grade 100% methanol (Product # 34860) was purchased from Sigma-Aldrich (St. Louis, MO). Both 0.1% trifluoroacetic acid (TFA) in acetonitrile (Product #LS121-4) and 0.1% TFA in water (Product #LS119-4) were purchased from Fisher Chemical (Fair Lawn, NJ).

### Heat and freeze-thaw conditions

Naloxone hydrochloride ampoules were thermally stressed to either heat or freeze-thaw conditions on a daily basis for up to 4 weeks. *Heat*: Ampoules were placed in an 80 °C temperature-controlled oven for 8 h and transferred to room temperature for 16 h. *Freeze-thaw*: Ampoules were placed in a − 20 °C freezer for 16 h and transferred to a 4 °C refrigerator for 8 h. The effects of these conditions on naloxone hydrochloride stability were evaluated on the following days: 1, 2, 3, 4, 5, 6, 7, 14, 21, and 28. To reduce the possibility of secondary reactions, exposure to thermal cycling proceeded with the day 28 ampoule and ended with the day 1 sample. Therefore, all ampoules were exposed for the same duration of time until the HPLC analysis. Ampoules stored at room temperature served as the experimental control.

### HPLC system and chromatograph conditions

Naloxone hydrochloride was detected and quantified using the Waters Alliance e2695 Separations Module (Waters Corporation, Milford, MA, USA) at a wavelength of 282 nm. The system was equipped with a Waters 2489 UV/Visible Detector (Waters Corporation) and a SunFire C18 column (Si-100 Å, 3.5 μm particle size, 4.6 mm i.d., 100 mm length; Waters Corporation), which was used to achieve chemical separation at 25 °C. The mobile phase consisted of a 70:30 ratio of 0.1% TFA in water to 0.1% TFA in acetonitrile, pumped at a flow rate of 1.0 mL/min. Each sample was evaluated with three independent 50-μL volume injections into the column. Data was collected and analyzed using Empower Software (Waters Corporation). Naloxone hydrochloride identity was confirmed using retention time matching with a reference standard (see Additional file 1: Figure S1 for a sample naloxone chromatogram). Naloxone hydrochloride peaks consistently measured at 1.5 min in both the reference standard and ampoule formulation.

### Naloxone hydrochloride calibration plot

A calibration plot was generated using the naloxone hydrochloride reference compound. A 500 μg/mL stock of naloxone hydrochloride dissolved in HPLC grade 100% methanol was filtered using a 0.45-um filter and diluted to the following concentrations: 200, 100, 50, 25, 10, 5, 3, and 1 μg/mL. Each sample was quantified by HPLC using the conditions specified above. The calibration plot was obtained by plotting the area under the curve (AUC) of each peak (*y*-axis) vs the corresponding concentration (*x*-axis). The calibration plot generated the linear function *y* = 7,602,326.35*x* − 8640.01 with *R*^2^ = 0.9997. The concentration of naloxone hydrochloride in thermally stressed samples was determined using the measured AUC (y) and solving for concentration (*x*).

## Results

**Fig. 1:**
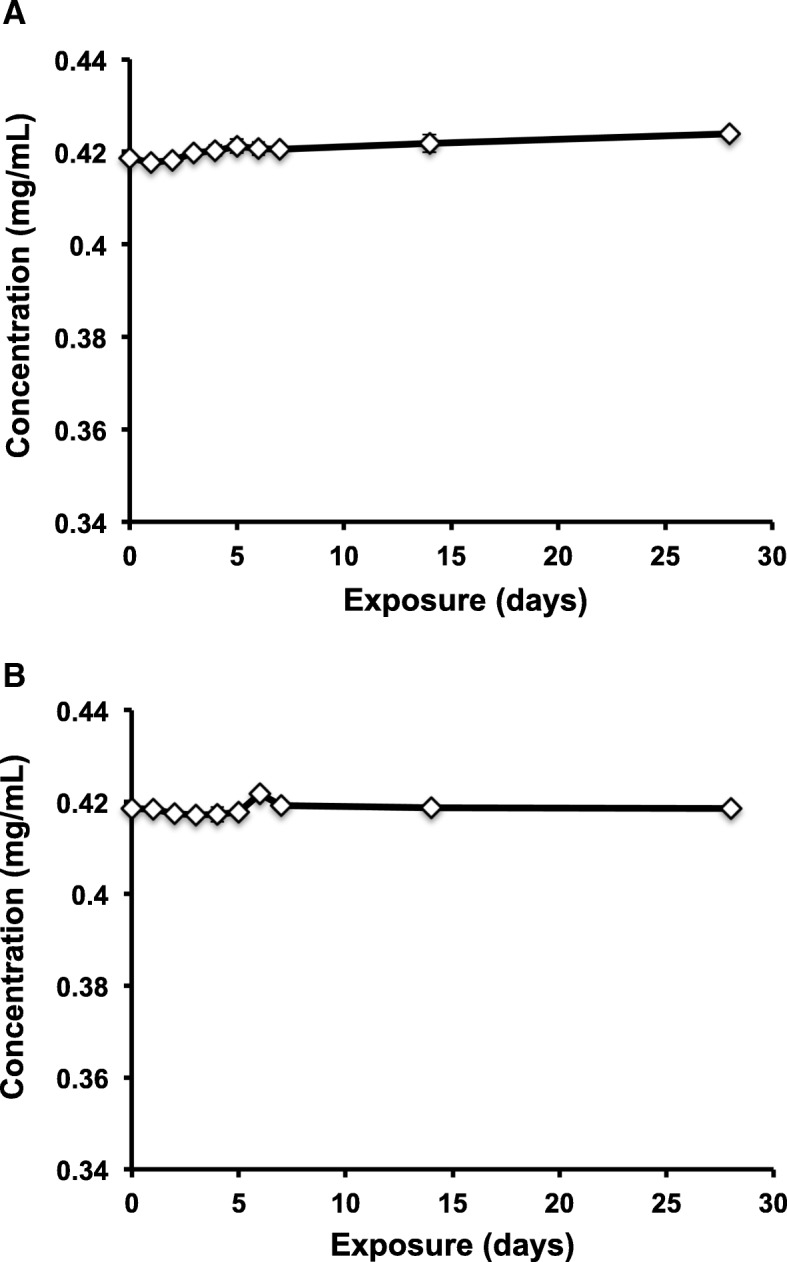
Naloxone hydrochloride maintains chemical stability following exposure to heat and freeze-thaw cycles. **a** Naloxone hydrochloride ampoules were exposed to 80 °C for 8 h daily followed by 16 h at room temperature, or **b** − 20 °C for 16 h daily followed 4 °C for 8 h for up to 28 days. Naloxone hydrochloride concentration was determined using HPLC at 282 nm and a calibration plot generated using a naloxone hydrochloride reference standard. Ampoules were exposed for the following number of days: 0, 1, 2, 3, 4, 5, 6, 7, 14, and 28. Naloxone ampoules stored at room temperature served as the experimental control (exposure of zero days)

**Fig. 2:**
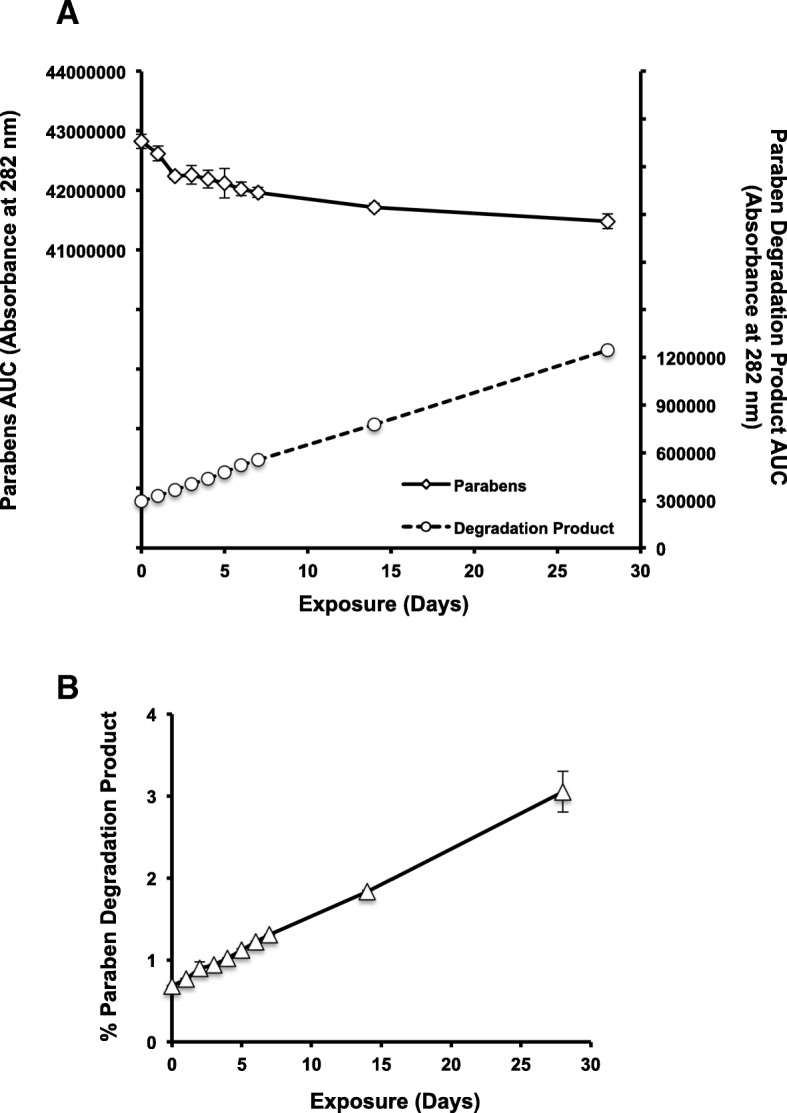
Degradation of the parabens correlates with duration of exposure to heat. The preserving agents found in naloxone ampoules are the parabens (methylparaben and propylparaben). **a** Chromatograms obtained from HPLC at 282 nm revealed an additional peak that increased in intensity (evaluated as area under the curve (AUC)) with duration of exposure to heat (indicated on the right axis). The left axis corresponds to the AUC for the parabens, which demonstrates a corresponding decrease. **b** The percent of paraben degradation product relative to total parabens as a function of time. Degraded parabens byproduct increases in abundance with increasing exposure to heat

When exposed to either heat (Fig. 1a) or freeze-thaw cycles (Fig. 1b), naloxone hydrochloride samples remained at comparable concentrations as ampoules stored at room temperature. In contrast, preservatives in the naloxone formulation, methylparaben and propylparaben, were subject to some degradation when exposed to heat. Degradation correlated with the duration of exposure as total AUC of the paraben chromatogram peak decreased with increasing exposure to heat (Fig. 2a). Likewise, the AUC of the paraben degradation product chromatogram peak increased with increasing exposure to heat (Fig. 2a). After 4 weeks, 3% of paraben degradation product was detected with 97% of total parabens remaining unchanged (Fig. 2b).

**Fig. 3:**
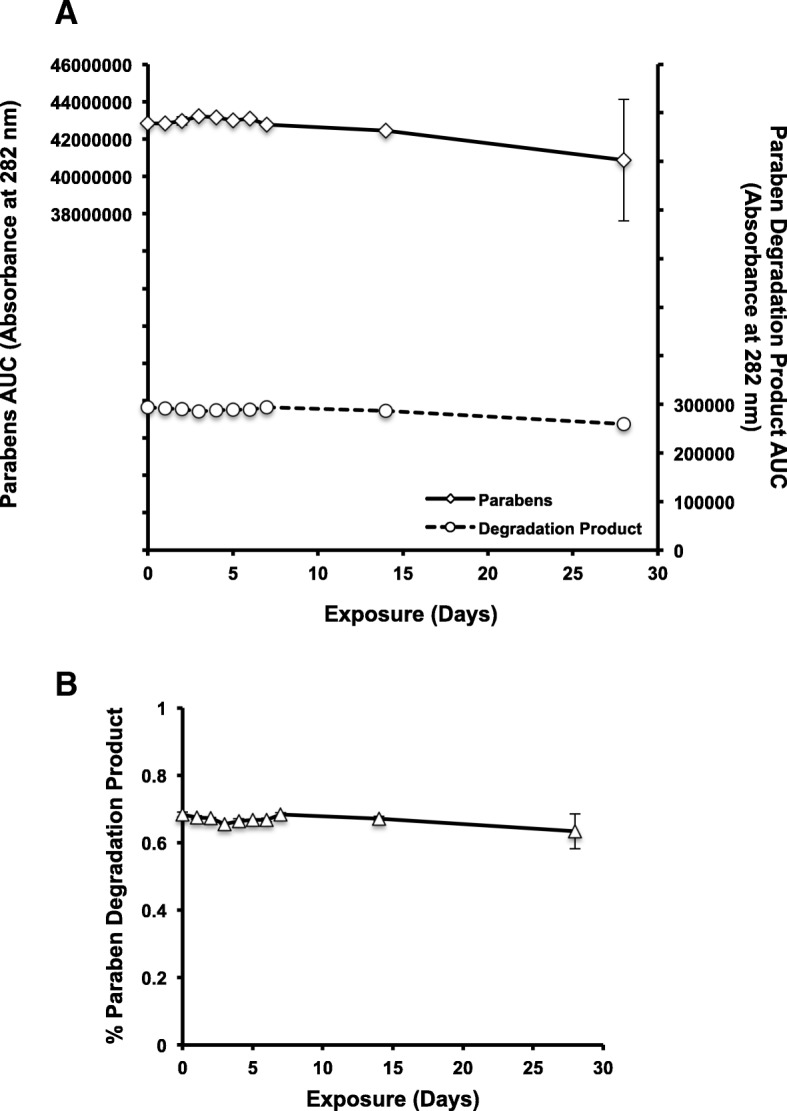
Parabens are not affected by exposure to freeze-thaw cycles. **a** The concentrations of both total parabens and the paraben degradation byproduct remain unchanged (evaluated as AUC) after 28 days of exposure to freeze-thaw cycles. The left axis corresponds to the AUC for the parabens whereas the right axis corresponds to the AUC for the paraben degradation product. **b** The percent of paraben degradation product relative to total parabens as a function of time. The degraded paraben byproduct concentration remained the same after 28 days of exposure to freeze-thaw cycles

No degradation was observed for the parabens under freeze-thaw conditions (Fig. 3a/b); however, two observations suggest changes in ampoule composition. After approximately 1 week of freeze-thaw cycles, visible white precipitate formed within the ampoules. In addition, although ampoule contents remained in liquid form following exposure to − 20 °C, contents began to freeze after 1 week of freeze-thaw cycling. These physical changes suggest that freeze-thaw cycles may be altering naloxone ampoule contents; however, the naloxone itself remains stable.

## Discussion

In Canada, drug manufacturers must complete stability testing as outlined by the ICH guidelines. Rarely do these guidelines address temperatures exceeding 40 °C or below 5 °C. In addition, research studies evaluating drug stability in extreme environments are few and far in between, especially those addressing the impact of freeze-thaw cycling. Therefore, this study was designed to address conditions that may be encountered by individuals in possession of a THN kit. We assessed the impact of daily exposure to either heat (80 °C for 8 h followed by room temperature for 16 h) or freeze-thaw cycles (− 20 °C for 16 h followed by 4 °C for 8 h) on naloxone hydrochloride stability. Regardless of duration and exposure to either condition, no detectable reduction in naloxone hydrochloride concentration was detected by HPLC, demonstrating drug stability under these conditions.

The stability of naloxone hydrochloride exposed to thermal stresses has been investigated by several studies. However, findings have been variable due to inconsistencies in experimental design, duration of exposure, and methodology. For example, older studies have found that naloxone remains stable under thermal stress whereas newer studies demonstrate heat-dependent degradation [12, 14–16]. In the earliest study, naloxone hydrochloride was stored in a white metal shed to simulate a paramedic vehicle parked during the summer months in Tucson, Arizona for 4 weeks [16]. Temperatures recorded ranged between 26 and 38 °C, which is marginally higher than the recommended storage conditions. These temperatures also fall within the temperature range recommended for stability testing according to the ICH guidelines. Considering these factors, it is not surprising that naloxone hydrochloride did not exhibit any changes in stability. It is, however, important to note that this study was conducted prior to the release of the ICH guidelines. Several years later, Johansen et al. exposed naloxone hydrochloride to − 20 °C, + 70 °C or fluctuating between the two temperatures [12]. Although the study assessed greater thermal extremes, the total exposure time was limited to only 16 h within a 48-h study period. These conditions had no effect on naloxone hydrochloride stability; however, it would have been insightful to study these effects over longer durations of exposure.

In contrast with these findings, newer studies have found that naloxone hydrochloride exhibits temperature-dependent degradation. Gammon et al. exposed naloxone hydrochloride to fluctuating temperatures of − 6 °C and + 54 °C every 12 h, and concentration was measured weekly for up to 4 weeks using HPLC [14]. By the end of 4 weeks, 89.62% of naloxone hydrochloride remained relative to the original concentration. This ~ 10% reduction in concentration was statistically significant. Despite the observed reduction in naloxone concentration, it is likely not clinically significant as the acceptable standards for manufactured and compounded drugs fall between 90 and 110% of the stated concentration [17, 18]. This study also possesses several limitations. First, the study did not contain a control ampoule stored at room temperature. Second, it appears that only one naloxone ampoule was used for the duration of the study. Although a baseline concentration was measured prior to thermal exposures, the remaining contents could have been subject to consequences of humidity (e.g., evaporation, condensation) or oxidation. A standard calibration plot was not included in this report.

Armenian et al. also observed naloxone hydrochloride exhibits temperature-dependent degradation [15]. Naloxone hydrochloride ampoules were subject to several conditions (*sustained* temperatures of − 20 °C, + 45 °C, or alternating weekly between the two) for 1 month. The most degradation occurred in the presence of heat with only 39% of the original concentration remaining after 4 weeks. Several factors may explain the degree of degradation observed. First, although ampoules were subject to moderate heat, the exposure was sustained for the duration of the study. Second, only one vial was used per thermal condition, which was sampled weekly. Therefore, the humidity and oxidation may have contributed to the extent of degradation. It is also important to note that an experimental control was not included in the study. Since HPLC was used to identify and quantify naloxone in this study, further characterization of degradation products by liquid chromatography-mass spectrometry (LC-MS) would have been useful in their overall analysis.

The limitations described above, in addition to other factors, were considered for our experimental design to reduce the impact of confounding factors. For example, no ampoules were tampered with until the end of the study where samples were run simultaneously via HPLC. This eliminated any impact that humidity or oxidation may have had on naloxone stability. Since all samples were run on HPLC at the same time, other external factors (e.g., column temperature) could also be controlled. Each sample was also measured in triplicate readings to ensure homogeneity of ampoule contents. Importantly, our study also included control ampoules, which were stored according to the manufacturer’s storage conditions.

However, this study is not without its limitations. First, we attempted to emulate conditions that would be generally encountered upon vehicle storage in winter or summer months. However, these conditions vary considerably. Ideally, assembled THN kits should have been stored in a car parked outside in the winter or summer months. Depending on the location within Canada, internal vehicle temperatures may differ. Temperature extremes may also be dampened by the THN kit housing and the location of placement within the vehicle. Second, although ampoules retain naloxone concentrations following thermal stress, we did not make any functional assessment of the naloxone samples (e.g., binding affinity to opioid receptors) in vitro or in vivo. Third, although ampoules were not tampered with until the end of the study, we still cannot eliminate secondary reactions or degradation that may occur from time of last exposure until the HPLC run. However, because ampoules were stored according to the product monograph during this time and all ampoules were exposed for the same duration of time, any effect would be negligible. Last, it would be useful to extend the duration of exposure beyond 1 month to assess how long naloxone hydrochloride can tolerate these conditions before the compound begins to break down.

## Conclusions

This study is the first in Canada to assess the practical impact of heat and freeze-thaw cycles on naloxone hydrochloride stability. Our findings suggest that exposure of naloxone hydrochloride ampoules to temperatures up to 80 °C or daily freeze thaw cycles for 1 month appear to have no impact on its stability. Therefore, naloxone hydrochloride ampoules should remain efficacious should THN kits be temporarily stored outside of the recommended storage conditions. Despite naloxone’s stability, and because the clinical implications were not evaluated, pharmacists should continue to counsel patients on the importance of appropriate naloxone storage to ensure optimal efficacy. Furthermore, if any signs of precipitate become visible in the ampoule, the ampoules should be replaced. However, the naloxone itself remains stable in solution, and injection of such a product is warranted to reverse a life-threatening opioid overdose.

## Additional file

Additional file 1: A sample naloxone chromatogram. (TIF 1072 kb)

## Abbreviations

AUC: Area under the curve
HPLC: High-performance liquid chromatography
ICH: International Conference on Harmonization of Technical Requirements for Registration of Pharmaceuticals for Human Use
LCMS: Liquid chromatography-mass spectrometry
NAPRA: National Association of Pharmacy Regulatory Authorities
OTC: Over-the-counter
TFA: Trifluoroacetic acid
THN: Take home naloxone
