# Supplementary figures and images for: The effects of heat and freeze-thaw cycling on naloxone stability
Source: Harm Reduct J. 2019 Feb 27;16:17. doi: 10.1186/s12954-019-0288-4 (PMC6391798; doi:10.1186/s12954-019-0288-4)

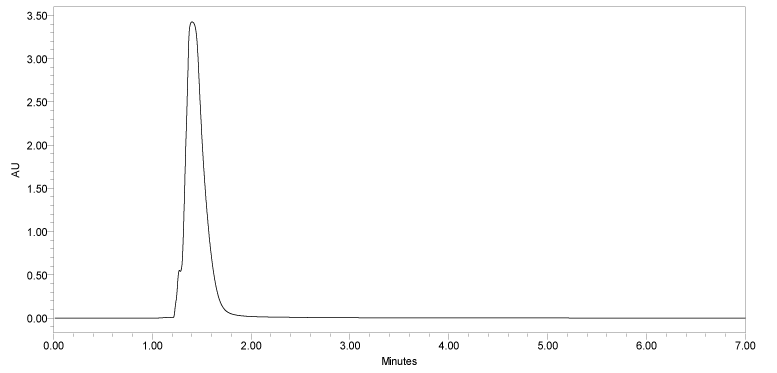

Supplement: Supplementary file 1 — A sample naloxone chromatogram. (TIF 1072 kb) [file 12954_2019_288_MOESM1_ESM.tif]
